# Persons with Haemophilia in Sweden- Experiences and Strategies in Everyday Life. A Single Centre Study

**DOI:** 10.1371/journal.pone.0139690

**Published:** 2015-10-02

**Authors:** Elisabeth Brodin, Katharina S. Sunnerhagen, Fariba Baghaei, Marie Törnbom

**Affiliations:** 1 Institute of Neuroscience and Physiology- Rehabilitation Medicine, Section for Clinical Neuroscience and Rehabilitation, Sahlgrenska Academy, University of Gothenburg, Gothenburg, Sweden; 2 Department of Medicine/Hematology and Coagulation, Coagulation Centre, Sahlgrenska University Hospital, Gothenburg, Sweden; 3 Sunnaas Hospital, Oslo University, Oslo, Norway; 4 Department of Social Work^,^ University of Gothenburg, Gothenburg, Sweden; University of Pennsylvania School of Medicine, UNITED STATES

## Abstract

**Introduction/Aim:**

Haemophilia is caused by deficiency in coagulation factor VIII or IX. Treatment with the missing coagulation factors has been available in most developed countries for several decades. The aim was to explore the experiences of adults living with severe or moderate haemophilia and their coping strategies at a single centre in Sweden.

**Method:**

The interview study had a qualitative empirical approach and was analyzed on the basis of the method empirical phenomenological psychology. The sample included 14 participants, mean age 42 (19–80 y), who met the inclusion criteria and to saturation of information. *Results*: General characteristics were; All were satisfied with and grateful for access to medication. An acceptance of the disorder and willingness to live a normal life was identified among all participants. They were all content with the care provided by Haemophilia Treatment Centre (HTC) and felt supported by its multidisciplinary team. Four typologies were identified; Protective adults and assertive children during up-bringing, finding a role in social context, symptoms and treatments, fear of limited resources in the future. Task-, emotional- and avoidance coping strategies were seen in the interviews. The most prominent coping strategy was task oriented.

**Conclusion:**

This interview study with Swedish PWH shows that they strive for normality and adaptation in social activities throughout life finding their own niche. The PWH expressed the importance of knowledge and support from the comprehensive medical team at HTC and therefore it seems important to continue comprehensive medical care at HTC in order to follow-up the haemophilia persons regularly.

## Introduction

Haemophilia is an X linked congenital disorder which is caused by deficiency in the coagulation factor VIII (haemophilia A) or coagulation factor IX (haemophilia B)[1]. Prophylactic treatment with coagulation factors has been available in most developed countries in recent decades [2, 3]. Studies[4–6] showed benefits of prophylaxis treatment from an early age but the medical costs for this treatment are discussed and many countries can´t afford it[3, 5].

Previous interview studies reported improved quality of life for adults with haemophilia due to the benefits of factor replacement therapy as compared to the time before factor replacement was readily available[7], that the bleeding disorder had an impact on education, work, social activities and family life of affected individuals [8], and that the disorder was always present in their life and the home treatment influenced everyday life [9]. These results were confirmed by a study of the patients perspective for understanding the impact of haemophilia symptoms and treatments on their Health Related Quality of Life [10]. To get a more profound understanding of how Persons with Haemophilia (PWH) experience their life situation and how they cope with their disability seems to be important knowledge for health professionals and others involved.

The aim of the present study was to explore the lived experiences and coping strategies of adults with severe or moderate haemophilia at a single centre in Sweden.

### Participants and Methods

The participants were recruited from the Haemophilia Treatment Centre (HTC) at Sahlgrenska University Hospital, Gothenburg (one of three HTC in Sweden). The selection criteria were as follows; age ≥18 years, having haemophilia A or B, severe or moderate form; all should be able to have an interview in Swedish; there was a wide age span and participants lived in a wide variety of places within south west Sweden. The total eligible subjects when the study was performed were 42 PWH, Table 1. Of these three had language difficulties, one was seriously ill, one had unknown address, two had serious social problems; one had moved to another country, and three were never included.

**Table 1:** Demographics of all adult patients at the HTC Sahlgrenska University Hospital beginning of 2012.

		Late onset	Early onset
**Individuals with haemophilia, all male (n)**	**42**	**15**	**27**
**Mean age, years (range)**	**39 (19–80)**	**59 (47–80)**	**29 (19–44)**
**Haemophilia A (n)**	**34**	**11**	**23**
**Haemophilia B (n)**	**8**	**4**	**4**
**Haemophilia severe form (n)**	**31**	**10**	**21**
**Haemophilia moderate form (n)**	**11**	**5**	**6**

n = number

Therefore an invitation letter was sent to 31 subjects by mail. Those who wanted to participate (17 persons) replied by returning written informed consent. Fourteen PWH with a mean age of 42 years (19–80 y) that confirmed participation were interviewed (convenience sample). When we had interviewed fourteen persons no new aspects appeared. The team assessed that enough information was gathered for the purpose of the study [11, 12] and therefore, three persons were not interviewed since saturation was achieved. [13]. The participants were divided into two age groups based on when they had access to factor replacement treatment; a late onset group, older (born 1931–1964, n = 7) and an early onset group, younger (born 1971–1992, n = 7). The late onset group started treatment as teenagers or as adults (on demand or prophylaxis) while the early onset group got factor concentrates during their upbringing[14–16]. The participants chose the time and place for their interview and completed a demographic questionnaire regarding gender, age, occupation, marital status and concomitant diseases. Each interview lasted 1–1.5 hours. During the spring of 2012 all individual interviews were performed by 1 of the 2 trained interviewers [17] but without specialist knowledge of haemophilia. The last author performed half of the interviews and had no clinical experience with haemophilia.

Ten interviews took place at the participants´ homes and four interviews in other locations. The interviews were performed supported by an interview guide with open-ended questions such as; “How do you experience living and dealing with haemophilia?” “How does haemophilia influence your life situation now and during your childhood?” “How do you experience your health care?”

The interviews were recorded with a digital tape recorder and transcribed word by word by clerks. No repeat interviews were performed and no transcripts were returned to participants.

The interview study had a qualitative empirical approach and was analyzed on the basis of empirical phenomenological psychology (EPP) by G. Karlsson [11], Table 2. Coping strategies were identified according to Lazarus[18]

**Table 2:** The five steps for the analysis according to empirical phenomenological psychology (EPP) by G. Karlsson (11).

Analysis steps	Content
**1. Good grasp**	Perusal of all the transcribed interviews to understand the whole
**2. Meaning units**	Classification of the interview text in meaningful units where a shift in meaning was identified in accordance to the purpose of the study
**3. Eidetic induction**	Eidetic interpretation was done from the participants everyday language to find the characteristics of the phenomena in the narratives by the participants
**4. Situated contexture**	Summary of the meaning units into a whole in order to describe how the phenomenon is lived and what the phenomenon is
**5. Finally: result in general characteristics and typology**	First, a specific description for each interview to compare thereafter the detailed interview subtitles with each other to achieve a general or a typical description of the phenomenon

The analyses were performed by the first and last authors. The first author, registered physiotherapist and PhD-student, did not interview because of clinical knowledge about the PWH participants. The participants knew about the involvement of the first author.

The results were presented in terms of experiences of the phenomena and the strategies used for coping with them. Experiences and coping strategies that all articulated were presented as general characteristics. Secondly, experiences and coping strategies present but not in all interviews were presented in terms of typologies. The themes are exemplified by quotes from the participants and if clarification is needed it stands inside a bracket.

The study was approved by the regional Ethical Review Board at the University of Gothenburg.

## Results

The demographics are seen in Table 3.

**Table 3:** Demographics of the participants and the PWH who declined participation in *italic*.

	Participate	Late onset	Early onset	*Declined*	*Late onset*	*Early onset*
**Individuals with haemophilia, all male (n)**	14	7	7	*14*	*3*	*11*
**Mean age, years (range)**	42 (19–80)	57 (47–80)	27(19–41)	*35(20–72)*	*63(50–72)*	*27(20–35)*
**Haemophilia A (n)**	10	5	5	*12*	*1*	*11*
**Haemophilia B (n)**	4	2	2	*2*	*2*	*0*
**Haemophilia severe form (n)**	10	4	6	*12*	*2*	*10*
**Haemophilia moderate form (n)**	4	3	1	*2*	*1*	*1*
**Marital status (n)**						
** Married**	6	5	1			
** De Facto**	2	1	1			
** Single**	6	1	5			
**Working (n)**	6	2	4			
**Studying (n)**	3	0	3			
**Sick-leave (n)**	2	2	0			
**Disability pension (n)**	3	3	0			
**Retired (n)**	2	2	0			
**Concomitant diseases (n)**	6	4	2			

n = number and declined participation in *italic*

Concomitant diseases were hepatitis, diabetes mellitus, kidney disease, hypertension, psoriasis arthritis, kidney stones, pollen allergy, depression, and attention deficit hyperactivity disorder (ADHD).

### General characteristics among all participants

All PWH were satisfied with and grateful for access to medication. They reported limited participation in athletics at school, especially group activities such as soccer and ice-hockey. All participants had been employed during adulthood. All stated that their closest colleagues at work knew about their condition. They accepted being PWH and said, for example that they; wanted to live a normal life, adapt to and cope positively with the disorder, and they might have to endure pain when performing activities. All PWH were satisfied with the health care provided by HTC and found the multidisciplinary team to be competent. They felt supported by the team around the clock and described good communication with staff. Also, accessibility by phone and frequency of contact were satisfactory.

### General characteristics for the late and early treatment onset group

The late onset participants, who had no access to prophylactic factor replacement during childhood, experienced limitations with physical activities during their childhood and had to give them up due to risks for bleeds.

The early onset participants felt that they were able to live an active lifestyle, very close to that of the unaffected population because of the treatment with factor concentrates.

### Typology I: Protective adults and assertive children during up-bringing

During childhood many PWH experienced anxious parents and cautious teachers and some of them felt over-protected, especially by mothers.

......."I lived in a room lined with foam rubber....” (code 1)

......." (my parents told me) don´t go out and don´t play with other children” (code 8)

The older participants sometimes felt that their parents were exhausted because of their need for care. One person moved from his parental home to reduce the burden on his parents.

The younger participants did not want to be treated differently compared to their peers. For example, a child wearing a helmet could be singled out by others. A way to cope with this situation could be that the PWH asserted himself with tough activities.

### Typology II: Finding a role in social context

#### Family, friends and social network

Family and friends knew about the disorder and what to do if there was an acute bleed. Several participants said they did not feel treated differently by their social network because of haemophilia.

Some said their social network had been supportive during their childhood. Playing with other children could be hindered due to bleeds. A PWH could find a way to participate in a game by finding a role that was not physically challenging.

……….“you took on a role in games (sport) where you could be the umpire/referee instead” (code 2)

Others said they had a smaller social network as adults than during their teens but had good relationships with relatives.

Some of the younger PWH told that they could perform all possible activities and therefore their friends did not treat them differently despite knowing about the disorder.

#### Education

Some of the older PWH had been absent from school because of bleeds and hospital admissions and missed education periodically and had to complete their education as adults. Individuals within their social network sometimes acted as teachers for PWH when they did not attend school. One was forced by authorities to accept special job training because of haemophilia. Some younger PWH actively chose education that was suitable considering their disorder, such as a university education as a way of avoiding physical work.

#### Work

Some PWH with physical demanding work had been forced to change occupation or study because their bodies did not cope. During rehabilitation PWH often were given special work tasks.

........“I have had real problems with my elbow (tennis elbow). I have had to stop working as a cook. Next autumn I will likely start some new job training”. (code 12)

One also felt discriminated against as PWH are not allowed to join the Swedish Armed Forces and thought this is a waste of resources.

Some felt relief at being able to work part time, with governmental financial support, due to their haemophilia arthropathy.

One of the older PWH had an experience of employment discrimination; he had been refused a job due to his haemophilia diagnosis. Another PWH was unemployed for more than a decade. He later got a position with wage subsidies. Some could not achieve their work ambitions because of their disorder. It had been hard for some of the older PWH to change occupation because of haemophilia arthropathy. Another example was not being able to get a health certificate.

All younger PWH worked or studied full time and they felt capable of working at that level. One, with a physical demanding job, stated his colleagues did not understand his invisible disability and were annoyed by his sick leave. One said haemophilia made him take precautions when working where he could be hurt. One thought it was unnecessary during an interview to inform an eventual future employer about haemophilia if he/she did not ask.

#### Leisure

Interests adapted to the disorder included involvement with disability organisations and cultural activities. A strategy to handle the risk for bleeding was to find a balance between physical activity/playing and inactivity.

…………“My interests are rather calm/sedate. I am keen on computers and that is also what I’m trained in.” (code 5)

Some felt frustrated that they could not perform sport activities due to joint damage.

Several PWH could not participate in their children´s physical activities, although they wanted to.

### Typology III: Symptoms and treatments

#### Bleeds and pain

Older PWH experienced bleeds and pain influenced their early lives to a great extent. Parents, at the time unaware that acetylsalicylic acid increased the bleeds, gave them this medication. Several had long periods in hospital because of serious bleeds.

“Pain… and I remember how we (participant and his brother who was also ill) screamed and cried for days with no solution, no help” (code 8)

Participants also experienced severe epistaxis/nose bleeds.

One had blood transfusions from his father during bleeding episodes. Bleeds also resulted in anaemia.

#### Joint function and arthropathy

Limitations of joint function in extremities influenced their ability to climb stairs, walk and carry and one person mentioned he was in a wheelchair during childhood due to arthropathy.

Some had also experienced joint surgery. The older participants stated that they had access to factor replacement nowadays. Because of pain and haemophilia arthropathy they needed medication for pain-relief and synovitis.

#### Factor replacement treatments

A few individuals felt discomfort when injecting the factor concentrate. Instead, they decided to take medication on demand. One of them suffered because of this strategy, he misinterpreted his symptoms as an inflammation and failed to appropriately medicate and bled more than might have otherwise been the case. When interviewed, some had stiffness and pain in spite of regular prophylaxis. These conditions were more severe when regular prophylaxis was missed by the patients. Some said that the inconvenience of having haemophilia was the morning ritual of injections.

A positive attitude to prophylaxis treatment with factor VIII or IX was expressed by some PWH. It was obvious that participants had knowledge of the effects of medication. When planning for a risky activity, sports and visit with a dentist, an extra prophylactic dose of factor concentrate was taken.

“It is really nothing (the morning ritual) compared to not having access to medication” (code 29)

Most of the younger PWH had regular prophylaxis treatment and no bleeding symptoms during their childhood. One person was afraid of injections as a child, but could inject himself nowadays due to training by the haemophilia nurses at hospital. Problems with inhibitors against factor VIII or IX during childhood were experienced by some PWH. The ones successfully treated expressed they were lucky and could live a good life again.

Some individuals had hepatitis C as a consequence of previous treatment. They found treatment of hepatitis C to be tough. There was fear of getting HIV and the wait for blood test results was terrifying.

#### Balance between exercise and rest

To prevent bleeds, physical therapy, individual exercise programs, rest, medication and “common sense” were mentioned as strategies. To struggle and fight against the disorder and to rise after crises were mentioned as strategies for acceptance in the face of symptoms and the treatment required in order to live a normal life.

PWH were content with follow-ups of joint and muscle function and specific advice about exercise.

#### Haemophilia Treatment Center, HTC

Some said that as a patient you have to inform care givers, not being specialists in bleeding disorders, about haemophilia and the importance of consulting the HTC. Several PWH found the HTC to be advocates for them in dealings with other units of Health Care, but especially in the old days. The HTC care was considered to be better in the old days but is still good enough.

The staffs were experienced to be more active with follow-ups in the early days and some participants missed retired competent staff.

One wanted more specific national guidelines for haemophilia treatment based on research approved by the National Board of Health and Welfare. He was keen on the same treatment and care at all HTC in Sweden. The staff should reveal their knowledge about results from research and also put them into practice at annual follow-ups.

*"*........ What they (the staff) should check for at the annual visit are those things that have been agreed to (in the guidelines) and I can make sure I get what is actually agreed to.*"* (code 13)

In an emergency or within the Primary Health Care system, especially in the countryside, it might be hard to convince staff of the importance of contacting HTC for proper care. Some were still content with the Primary Health Care system.

#### Authorities

Among the younger PWH, one stated that it is currently not possible to get life insurance if you have haemophilia, but that considering how effective treatment is that it should be. He suggested it should be a task for the National Board of Health and Welfare, in cooperation with the health care system, to make it available.

### Typology IV: Fear of limited resources in the future

#### Subsidized medication

Among older PWH some talked about how lucky they were living in Sweden because of the available medical treatment, which is subsidized. Several PWH among the younger group explicitly said that the medication is very expensive and should be used in an efficient way but not excessively. Because of medication some said they were the first generation to have an almost normal life expectancy. One person had worries that children could be prioritized for prophylaxis and not older PWH if resources became limited in the future. Conversations with people from outside Sweden have made participants aware of the privilege they have when it comes to subsidized medicine, as compared to some other countries.

One said that it would be more expensive for society if PWH were unable to work; it would be cost-effective to give them medication. Some were worried that younger PWH could get physical symptoms if they do not use their medication as a prophylactic treatment.

#### Decreased resources

Worries about cuts to health care were pronounced among the older PWH. Some said that organizational changes and doctors under pressure sometimes influenced accessibility to health care. A few said they did not want to be a burden for their relatives but some were practically supported by their family, especially those with haemophilia arthropathy.

Different types of rehabilitation within health care have diminished over time. One PWH argued that rehabilitation also has a psycho-social function and is therefore important. The same person said that there is a need for rehabilitation abroad in an agreeable climate but is not subsidized by health care and everyone cannot afford it.

Another participant is a member of a committee of a disability organization working with haemophilia topics for older people. A greater understanding of specific geriatric conditions from the HTC team was requested as many PWH are ageing. He was concerned that older PWH won´t be able to get adequate treatment within geriatric units in the future, especially in the countryside.

……."When you come to an aged care facility are there people there who can give you your injections?” (code 2)

In Table 4 the typologies are summarized.

**Table 4:** Summery of typologies.

Typology	Experiences; Late onset/Early onset	Strategies; Late onset/Early onset
**I: Protected adults and assertive children during up-bringing**	...."the teacher in my new class told my mates to be careful with me and I found it distinguishing” (code 15)**/**....”I lived in a room lagged with foam rubber” (code 1)	.…" I became disorderly and fought with all (code 15)**/** …."I looked at my mates when they were playing soccer and was offered the sport clubs tracksuit and participated” (code 1)
**II: Finding a role in social context**	...." I understood that I didn´t manage any longer to work full time” (code 15)**/**....“I had to stop work as a cook because of elbow pain” (code 12)	....“I took the role as a judge when the others were doing sport activities” (code 2) **/**…."Next autumn I think it will be some education”. (code 12)
**III: Symtoms and treatments**	…. “I have still problems with my joints that are painful (because of bleedings)” (code 20)**/ ….”** I have a crappy ankle (due to bleeding symptoms after a sprained ankle)” (code 30)	**…."** I went in for daily physical activities…..took walks outside” (code 15)**/** ….“It is really a trifle (morning ritual taking the medication) compared to not having access to medication” (code 29)
**IV: Fear of limited resources in the future**	…."When you come to an age care facility are there persons who can give you your injections?”…. (code 2)**/.**…"sometimes I worry that prophylactic medication is questioned (due to societal expenses)” (code 29)	.…" started a group in the Swedish Hemophilia Society to retain the interest of the elderly persons” (code 2)**/**

Examples for the PWH divided in late and early onset group

### Coping strategies in the study group

Task-, emotional- and avoidance coping strategies were seen in the material [18]. The most prominent coping strategy was task oriented. PWH could act in an assertive way when protected during up-bringing. They could plan and act to reduce symptoms and choose suitable education and work. They could also try to influence political decisions. The emotional coping strategies were positive attitudes to prophylaxis and inner struggles against the disorder, also rising after crises. Only one avoidance strategy was defined; not informing about being a PWH.

## Discussion

The aim of the study was to enhance knowledge about the lived experiences of PWH in Sweden and their coping strategies.

Overall, limited participation in athletics was reported. Swedish HTC recommend physical activity but not excessively. This is in accordance with the international guidelines. Despite prophylactic treatment with factor concentrates that allows exercise, a sports injury can cause bleeding complications for the PWH [19]. Both older and younger persons mentioned that they may have to endure pain as a consequence of their bleeding disorder if they get hurt in an activity or as a consequence of arthropathy. It has been described recently that pain has a social impact on quality of life [20].

Persons with haemophilia strove to live normally regardless of existing disabilities or not. All were aware of that pain could occur as a result of a haemorrhage. Impaired quality of life influenced by pain and arthropathy are seen among haemophilia patients when general (global) and disease specific questionnaires are used and are common in the physical domains [20–22]. The middle-aged and elderly PWH in a Swedish study, were found to have a lower quality of life,, especially in the physical domain, than a reference cohort of Swedes [21]. In a study from Austria by Hartl et al. [23] the results indicated that social status among adult PWH was not affected by their chronic disease and they were highly capable of coping with their disorder nowadays. The same study showed more unemployed and retired persons in the haemophilia group compared with the general population in Austria [23].

In contrast to these Austrian results [23] all PWH in this study had been employed during adulthood. In a recently published study from Sweden, 7% of the haemophilia population was unemployed [24]. In Sweden at the time for the study the unemployed rate was 6,4% in the population [25]. It seems that work and education for the Swedish PWH is comparable with the general population.

However, several persons had changed occupation during their working life when arthropathy had interfered with their work. Higher education was the solution for a sustainable working life among young PWH. Part time disability pension or wage subsidies were a relief that gave older PWH possibility to have a work situation they could manage. Only one interview showed discrimination related to work. Discrimination at school/work was much more pronounced in an English study by Barlow et al.[8].

The treatment maintains independency regarding the risk of occurred bleeds in terms of increased control related to activities. The prophylactic factor replacement treatment became available in the 1960s in Sweden[14]. This means that starting prophylaxis at an early age made it possible to live an almost normal life [26–28]. In this study, the older PWH had not had access to medication during childhood and therefore had got greater functional limitations as adults than the younger PWH. Many of them had one or more joints affected after bleeding episodes. Arthropathy is one of the most substantial symptoms of haemophilia if proper prophylactic treatment is not available or in the presence of inhibitors[29]. The benefits of replacement therapy with factor VIII or IX are also articulated in this study as in England and Finland [7, 9, 30]. Several PWH in this study expressed concerns about possible future limitations regarding treatment, if subsidies for prophylaxis were to be withdrawn in periods of financial cuts in health care. A Belgian study [31] also noted that the mental component in the quality of life assessments was greatly influenced by patients concerns and understandings of haemophilia-related financial issues.

Most of the participants also expressed that having haemophilia was a part of them in everyday life, something they must handle. They found a way to avoid the risks associated with bleedings. The whole group described these strategies for avoiding risk activities and these were in accordance with findings from studies by Beeton et.al.[7] and Peltoniemi et al.[9].

Like the Finnish study [9], the same limitations were described, such as not being able to enlist in the national army or manage to perform all wanted activities.

The PWH in our study felt their network was supportive, above all practically. It was also seen that parents could be affected by their children’s disorder. Barlow et al.[8] reported that parents´ working and economical life were influenced in a negative way.

Many of the younger participants had felt stigmatized during childhood due to their functional limitations and their needs to wear all sorts of protective clothing such as a helmet when playing and in sport activities at day-care and in school. Peltoniemi et al.[9] found PWH that felt bullied due to haemophilia and also had absences from school due to hospital visits which is in line with our findings. This study showed that several PWH strove for normality. The younger ones demonstrated this by climbing trees and play ice hockey when they were children. A Dutch study[32] showed that younger PWH had the same activities as their peers and a quality of life equal to the general Dutch population. Of those with a severe form of haemophilia, one third also participated in high risk sports [32]. The younger PWH, in our study who were without functional limitation, did not tell everybody about their condition. They did not want to be looked upon as the “PWH” that can´t do everything. Many had the childhood experiences of being considered incapable and wanted to get rid of it as adults.

Most of the participants in our study had good experiences from their contacts with the HTC and there were no findings about lack of trust, especially among younger PWH. In the general (global) quality of life questionnaire as EQ5-D or SF-36 there is no question about satisfaction with contacts with health care. For PWH in our study it seems valuable with a positive regular contact with HTC and this theme could be interesting to include in a disease specific questionnaire. Peltoniemi et al.[9] as well as Barlow et al.[8] found that PWH had a lack of trust in the knowledge and skills of the non-specialised health service staff and they felt that they were not supported and counselled enough. Our study showed that PWH needed to let the Primary Health Care staff know that it was important that the HTC staff be informed that the PWH had visited and received care at the Primary Health Care facility. They were mostly satisfied with the knowledge and support from the HTC. A few older PWH felt that nowadays they must be more responsible for their own health and were stressed about it. They felt the staff at the HTC was overwhelmed by work. These experiences are in accordance with what happens in healthcare in Sweden nowadays[33]. They experienced a need for more competence in the field of geriatric medicine within the HTC because of the growing number of elderly PWH.

An interesting idea found in our material was that some wanted more information about news from research from staff members at HTC, which we did not find documented elsewhere. In an Irish study by Goran et al a model was found to involve PWH as partners of the HTC in order to improve care and user experience [34]. In Sweden there is a user’s council that has annual meetings with the HTC team members where such issues are discussed.

The distribution of coping strategies in this study is similar to a study of PWH in the Netherlands [35]. The results in the present study describes how experiences can lead to specific coping strategies. However, it should be noted that in the present group there was a dominance of positive strategies.

The strengths with this study were the selection of both older and younger PWH, living in both cities and the countryside. The first author did not interview the participants because of longitudinal clinical contacts with the participants, but has been active during the period of analysis together with the last author who did half of the interviews. One limitation of this study was that we did not include persons with mild haemophilia and therefore experiences and coping strategies of this group cannot be articulated. The method used focuses on the experiences and coping strategies in relation to hemophilia. Other qualitative methods might have given other angles from the interviews.

## Conclusions

This interview study with Swedish PWH shows that they strive for normality and adaptation in social activities throughout life finding their own niche. The medication was praised as a precondition for being able to live an almost normal life. Thus some PWH felt stigmatized during childhood due to protective adults. They expressed the importance of knowledge and support from the comprehensive medical team at HTC. Among the older participants the need to take responsibility for their own health was a challenge and a stress factor. It seems important to continue comprehensive medical care at the HTC in order to follow-up PWH regularly.
